# Preserving Softness and Elastic Recovery in Silicone-Based Stretchable Electrodes Using Carbon Nanotubes

**DOI:** 10.3390/polym12061345

**Published:** 2020-06-14

**Authors:** Andrey Bannych, Sari Katz, Zahava Barkay, Noa Lachman

**Affiliations:** 1 Department of Materials Science and Engineering, Tel Aviv University, Tel Aviv 6997801, Israel; bannychandrey@gmail.com; 2 Department of Space Environment, Soreq NRC, Yavne 81800, Israel; sarik@soreq.gov.il; 3 Wolfson Applied Materials Research Centre, Tel Aviv University, Tel Aviv 6997801, Israel; barkay@tauex.tau.ac.il

**Keywords:** nanocomposites, multifunctional composites, soft sensors, mechanical properties, electrical properties, microstructural analysis

## Abstract

Soft electronics based on various rubbers have lately been needed in many advanced applications such as soft robotics, wearable electronics, and remote health monitoring. The ability of a self-sensing material to be monitored in use provides a significant advantage. However, conductive fillers usually used to increase conductivity also change mechanical properties. Most importantly, the initial sought-after properties of rubber, namely softness and long elastic deformation, are usually compromised. This work presents full mechanical and electro-mechanical characterization, together with self-sensing abilities of a vinyl methyl silicone rubber (VMQ) and multi-walled carbon nanotubes (MWCNTs) composite, featuring conductivity while maintaining low hardness. The research demonstrates that MWCNT/VMQ with just 4 wt.% of MWCNT are as conductive as commercial conductive VMQ based on Carbon Black, while exhibiting lower hardness and higher elastic recovery (~20% plastic deformation, similar to pure rubber). The research also demonstrates piezo-resistivity and Raman-sensitivity, allowing for self-sensing. Using morphological data, proposed mechanisms for the superior electrical and mechanical behavior, as well as the in-situ fingerprint for the composite conditions are presented. This research novelty is in the full MWCNT/VMQ mechanical and electro-mechanical characterization, thus demonstrating its ability to serve as a sensor over large local strains, multiple straining cycles, and environmental damage.

## 1. Introduction

Stretchable electronic applications, such as wearable electronics, soft robotics, personalized health monitoring, and sports performance monitoring, are mostly based on carbon-reinforced elastomers [1,2]. Being piezo-resistive, many of these materials have also been investigated as pressure-sensors [3,4,5,6,7,8] and strain-sensors [9]. Most focus among these conductive elastomers is concentrated on carbon nanotubes (CNT)-based conductive elastomers, as their superior conductivity and high aspect ratio allow for conductive networks at a lower volume fraction than the commercially available carbon black (CB)-based elastomers [1,3]. Indeed, elastomers such as natural rubber [3,10], styrene-butadiene rubber [3,11], and room-temperature vulcanized silicone rubber [4,12] have been successfully reinforced with multi-walled CNT (MWCNT). However, two problems hinder CNT-based elastomers from maturing into applicable technology. The first such problem is the high surface area of CNT which, despite enhancing mechanical and electrical contributions, also significantly increases Van-der-Walls interactions between the tubes, resulting in hard-to-break agglomerates which in turn deteriorate the mechanical properties of the composites, such as ultimate tensile strength and strain at failure, in some aspects even below the initial neat matrix [11]. Obtaining a homogeneous dispersion of the CNT in the matrix is thus fundamental for acquiring any desired properties [1]. Unfortunately, the most common practices of MWCNT dispersion are based on powerful solvents and sonication [1,12,13,14,15,16,17,18,19,20], techniques that are unsuitable for large volumes. Worth mentioning are the few works which have used pure mechanical methods such as ball-milling [5] and two-roll-mills [1,6,20,21], but those usually still required a pre-treatment, thus severely hindering the upscaling of the process to industrial levels. MWCNT-based vinyl-methyl silicone (VMQ), one of the high-end elastomers [22] has so far only been processed in solution [1,14,23].

The second problem, and one which is often overlooked, is the tendency of conductive fillers of any kind to increase hardness together with conductivity, due to the Payne effect [11]. Indeed, commercial conductive elastomers based on CB usually exhibit high hardness (50–70 Shore A), as well as low reversible deformation (~70% compression set—plastic deformation) [24,25]. This effect is unfortunate, as most of the aforementioned applications require the elastomer to be soft, compliant, and capable of high reversible deformation [2]. Nevertheless, very little work focuses on strain-sensing tests for compliance, and none test for hardness, the parameter affecting local deformations. Here, a scalable method for preparing a soft conductive vinyl-methyl-silicone composite rubber with MWCNT as the conductive filler is reported. Also presented are the material strain-sensing abilities—both electro-mechanical and aging induced strain sensing. Moreover, using morphological characterizations of the MWCNT dispersion, a mechanism for both the conductivity and the strain-sensing is proposed. The full mechanical and electrical characteristics are essential for the accurate assessment of the material performances as a sensor.

## 2. Materials and Methods

### 2.1. Materials

The matrix was commercial-grade mixtures of silicone rubber vinyl-methyl-silicone (VMQ) (Wacker Chemie AG, Munich, Germany), where 60% of the methyl groups on the silicone backbone have been replaced with vinyl groups. The hardness of the vulcanized VMQ as-is was 30 Shore A. The filler was a 10 wt.% commercial masterbatch of non-functionalized multiwall carbon nanotubes (MWCNT) produced via the catalytic chemical vapor deposition (CCVD) process, with average diameter of 9.5 nm and average length of 1.5 μm dispersed in vinyl-terminated silicone rubber mixture. The carbon purity in the masterbatch is 90%, and high resolution XPS measurements show 0.6% Oxygen on the MWCNT. BET surface area analysis of the MWCNT in the masterbatch was 250–300 m²/g. MWCNT were used as received.

### 2.2. Preparation of MWCNTs Filled VMQ Elastomers

**Figure 1:**
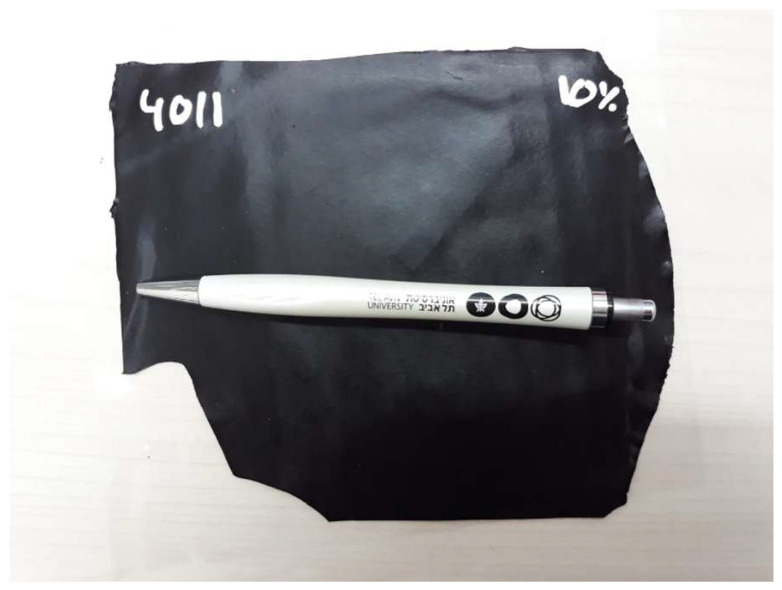
Vulcanized plate of VMQ with 10 wt.% of MWCNT. Pen to scale.

The MWCNT master batch previously mentioned was mixed with VMQ matrix to produce various weight percentage composites, ranging from the original 10 wt.% down to 1.25 wt.%. All mixtures were made by two roll mills, with 200 mm rolls in diameter and a ratio between turning speeds of 1:1.4, in room temperature, for half an hour. The peroxide-based curing system, dicumyl peroxide by the industrial name Perkadox BC-40B (AkzoNobel, Arnhem, The Netherland), was added in amount of 2.5 wt.% per new silicone mixture addition in each step of the dilution. The final composite mixtures were vulcanized at 170 °C for 10 min and then post cured for 4 h in 200 °C as control plate by compression molding in the mold with very precise cavity dimensions of 150 × 150 × 2 mm^3^. A neat VMQ matrix was prepared in the same manner as a reference. Figure 1 shows the resultant plate.

### 2.3. Mechanical and Electrical Characterization

Tensile tests specimens were cut from the vulcanized plate according to ASTM D412 standard, die D, using only one shot of the blade for each dumbbell to a total of 3 dumbbells. The stress/strain tests were performed at 500 mm/min at room temperature on a standard tensile Testometric™ (Rochdale, UK) machine, model M350-5CT equipped with a 500 N load cell and a mechanical extensometer with initial distance 25 mm between them. The changes in conductivity was also measured on this device while preforming the required elongation. The devices are calibrated yearly by the IAI Standards Laboratories Department.

The hardness was measured by a Durometer Shore A (SaluTron, Frechen, Germany), calibrated yearly by the IAI Standards Laboratories Department according to ASTM D2240, on each control plate stacked by 4 layers to create thickness of ~8 mm, as allowed by standard.

Compression set was measured according to ASTM D395-03 test method B in oven Heratherm OMH 60 (ThermoFisher Scientific, Waltham, MA, USA) by Dial Indicator calibrated yearly by IAI Standards Laboratories Department. 29 mm diameter and 12.75 mm high buttons (dimensions depending on material thermal expansion) were prepared and compressed between two plates down to height 9.51 mm (height of steel spacer, H_1_). The compressed buttons were heated in an oven for 175 °C and kept for 22 h. Afterward they were immediately released and rested at least 30 min before measuring their final height. By equation given in ASTM D395-03 test method B ([(H_0_ − H_2_)/(H_0_ − H_1_)] × 100) Compression set value was calculated. At least 5 specimens from each CNT concentration were measured.

The nanocomposite resistance was recorded with Agilent digital multimeter model 34401A (Agilent, Santa Clara, CA, USA) using 4-point probe method by modified ASTM D991 test probe (52-99-B305-0002) for measuring the volume resistivity of conductive elastomers.

### 2.4. Morphological Characterization

Quanta 200FEG ESEM (Environmental Scanning Electron Microscope) (Thermo Fisher Scientific, Eindhoven, The Netherlands) was used for the specimen morphological characterization under high vacuum conditions without conductive pre-coating.

In-situ tensile measurements in ESEM was performed using Kammarath & Weiss Tensile system (Kammrath & Weiss GmbH, Dortmund, Germany) (5000 N module). Strips, ~12 × 4 × 1 mm^3^ in dimentions, of sliced 10 wt.% MWCNT composites were tested, and images were taken at various elongation percentages up to ~100%—being the maximum step of the elongation.

### 2.5. Raman Characterization

Raman spectra were acquired by DXR2 (Thermo Fisher, Waltham, MA, USA) with a 532 nm laser excitation. Laser power at the samples was 5 mW.

## 3. Results and Discussion

### 3.1. Morphology and Dispersion

**Figure 2:**
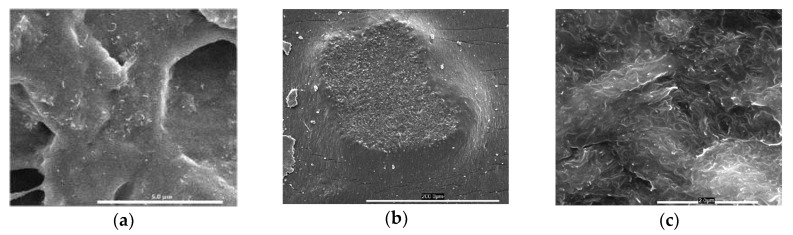
Representing SEM images of CNT/VMQ composites. (**a**) good CNT dispersion inside a CNT/VMQ composite specimen. (**b**) CNT agglomerate inside the composite. Note the matrix presence inside the agglomerate, and the ripples around it. (**c**) zoom-in into the agglomerate in figure b. Note that despite the high CNT concentration, all of them are encapsulated within a matrix.

SEM images (Figure 2) show a relatively good adhesion between the CNT and the VMQ matrix, as indicated by the lack of voids between the CNT and their surrounding matrix (most clear in Figure 2c). Also demonstrated is the tendency of CNT to re-agglomerate during the cross-linking after dispersion, as there were still many few-micron diameter agglomerates within the cross-linked rubber, but all of them contained some fraction of the matrix itself—indicating a high degree of matrix infiltration in between the CNTs. If those agglomerates originated from before the milling dispersion, we would have expected to see “naked CNT” with no surrounding matrix.

Note, however, that the agglomerates still manifest weak points: the simple difference in thermal expansion between VMQ (~4%/°C) and MWCNT (practically negligible) is enough to create internal stresses that micro-crack the close vicinity of these agglomerates. These cracks will affect the tensile strength of the MWCNT/VMQ composite.

### 3.2. Electrical and Mechanical Properties

**Figure 3:**
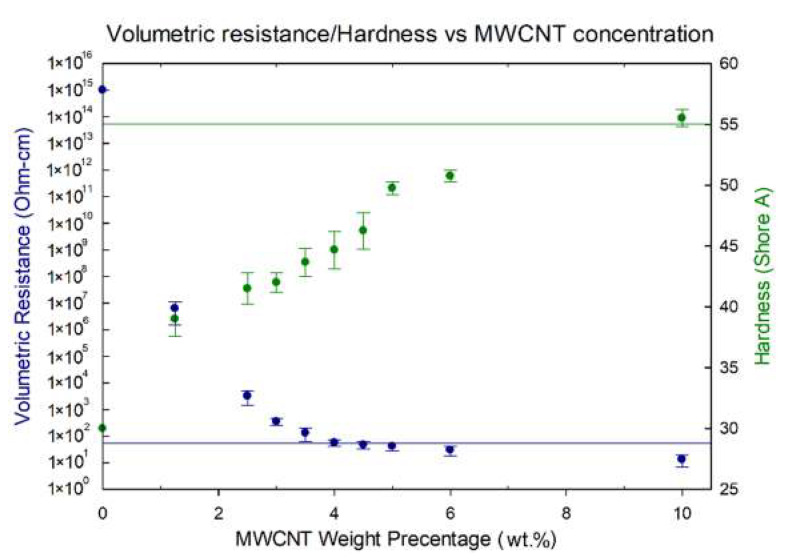
The volumetric resistance (Ohm-cm) and hardness (Shore A) of VMQ/MWCNT with respect to the MWCNT weight percentage. The horizontal lines on the graph mark the lowest hardness (top) and highest resistance (bottom) of the best commercially available carbon-black based conductive VMQ, i.e., the effective state-of-the-art. As can easily be seen, 4% MWCNT reinforcement meets the resistance of the state-of-the-art, while being significantly softer.

Unsurprisingly, increasing MWCNT concentration increases simultaneously the hardness of the rubber together with its conductivity (see Figure 3). However, both increases are non-linear, allowing an optimization of relatively high conductivity and low hardness.

The resistivity of pure silicone rubber drops exponentially with increased CNT percentage from little effect of 1.25 wt.% MWCNT and up to 4 wt.% of MWCNT added. Further addition of CNTs demonstrated no significant influence on the composite volumetric resistance.

This phenomenon fits nicely to the model of percolation threshold theory for rods [13]. First, because the observed conductive behavior is exponential, i.e., increased rapidly at first to reach an asymptote, as typical for CNT/composites [15,26]. Second, and more interestingly, the electrical measurements require time to stabilize, between 1 and 10 min to reach minimum value. This phenomenon is unique to CNT as conductive filler in rubber, as the electric force both assists in aligning the CNT and enhances the attractive forces between neighboring CNTs [26]. Since the rubber chains are flexible, the CNT can adjust their orientation and location to improve the conductivity of the network.

**Figure 4:**
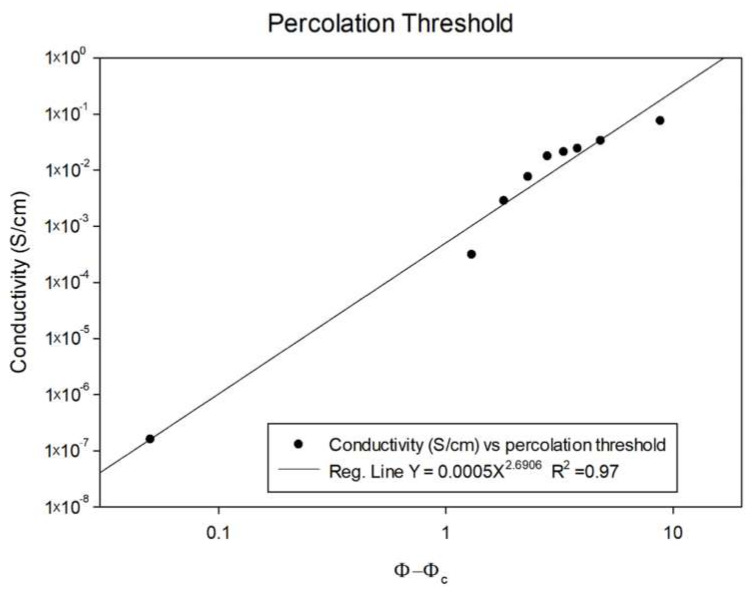
Calculating percolation threshold based on a best linear fit to experimental data.

The experimental data can be fitted to percolation power law of the shape σ = σ_0_(ϕ-ϕ_c_)^−t^ where σ is the measured conductivity (σ_0_ a proportion constant usually determined by the matrix parameters), ϕ the particles concentration, ϕ_c_ the critical concentration required for a conductive pathway (i.e., the percolation threshold), and t the critical exponent, usually attributed to tunneling effects [27] (Figure 4). Allowing the percolation threshold ϕ_c_ to be numerically adjusted for the best linear fit, the first conductive pathway should appear at 1.2 wt.% Indeed, the multimeter detection limit was crossed at 1.25 wt.% of CNT, as below that concentration, resistivity was too high to measure. Using Balberg expansion for rod-shaped particles [28]:

(1) $$\Phi_{c}=\frac{1}{2}\frac{D}{L}\frac{1}{\left(\frac{\Delta }{D}-1\right)}$$

where *D* is the rod diameter, *L* its length, and Δ the center-to-center distance between rods. The tunneling gap in that case can be calculated as:

(2) $$\Phi_{c}=1.2\;\mathrm{wt}.\%=\frac{1}{2}\frac{9.5\;\mathrm{nm}}{1500\;\mathrm{nm}}\frac{1}{\left(\frac{\Delta }{9.5\;\mathrm{nm}}-1\right)}\to \Delta =12\;\mathrm{nm}$$

Since the MWCNT average diameter in this work is ~9.5 nm, the tunneling gap is only slightly larger, allowing for a conductive path between neighboring CNTs.

As a side note, not adjusting the threshold for best linear fit results in a threshold of 0.4 wt.% MWCNT, indicating that approximately two-thirds of the MWCNT do not contribute to the network. As indeed can be seen under SEM—a large portion of the MWCNT is agglomerated in few-micron clusters, lowering the effectivity of densely-packed MWCNT participating in the conductive network. Thus, the corrections to the model should focus on the effective dispersion, rather than the dielectric constant of the matrix.

Unexpectedly, from the rule-of-mixture, hardness also increased non-linearly (see again Figure 3). Up to 5 wt.% of MWCNT, the hardness indeed increases linearly, but at that point, the slope decreases significantly, resulting in a much softer rubber than expected by continuing the early trend. The trend-shift indicated that the reinforcing mechanism of MWCNT in VMQ depends on more than just the average concentration of MWCNT in the matrix, demonstrating again the effect of dispersion and MWCNT/matrix friction on the overall mechanical properties.

**Figure 5:**
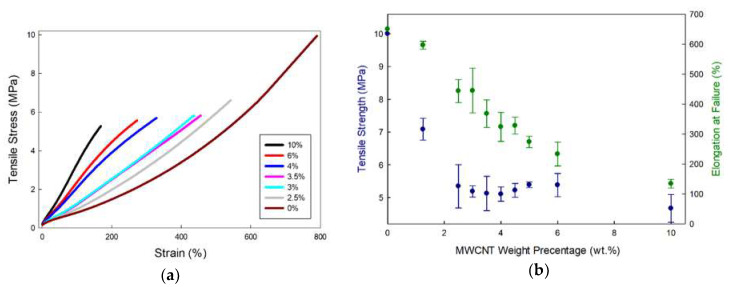
(**a**) stress-strain curves of various CNT concentration based VMQ; (**b**) the resultant tensile strength and strain at failure of these composites. For comparison, 10 wt.% In comparison, CB/VMQ has tensile strength of ~5 MPa and strain-at-failure of 250%.

**Figure 6:**
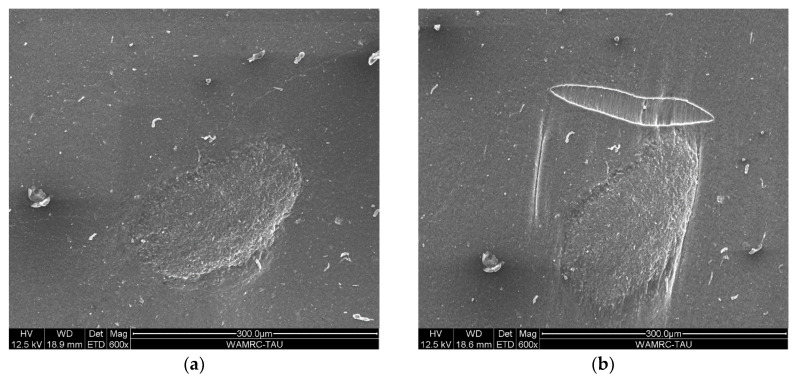
10 wt.% MWCNT/VMQ composite under tension in SEM. (**a**) an agglomerate before elongation. (**b**) the same agglomerate at 100% strain along the vertical axis of the image.

The mechanical properties under tension of relatively high concentration MWCNT/VMQ composite are very similar to those of state-of-the-art CB/VMQ (See Figure 5). The Young’s modulus increases with increased MWCNT concentration, indicating good adhesion. However, the addition of MWCNT leads to a reduction in the tensile strength. It is in fact quite common for MWCNT agglomerate to act as defects and stress-concentrators and lower tensile strength, even as they increase modulus [29]. Moreover, as MWCNT are much stiffer than the rubber itself, inhomogeneous dispersion means that MWCNT-rich areas elongate much less than MWCNT-poor areas near them, and the different restrictions cause inner shears resulting in fracture. Indeed, in-situ tensile tests performed inside the SEM (see Figure 6) show a tear not touching the agglomerate, but rather in its vicinity, at a relatively less reinforced rubber. This image is also an indication of the good adhesion between MWCNT and VMQ, as the agglomerate does not detach from the matrix upon stretching.

**Figure 7:**
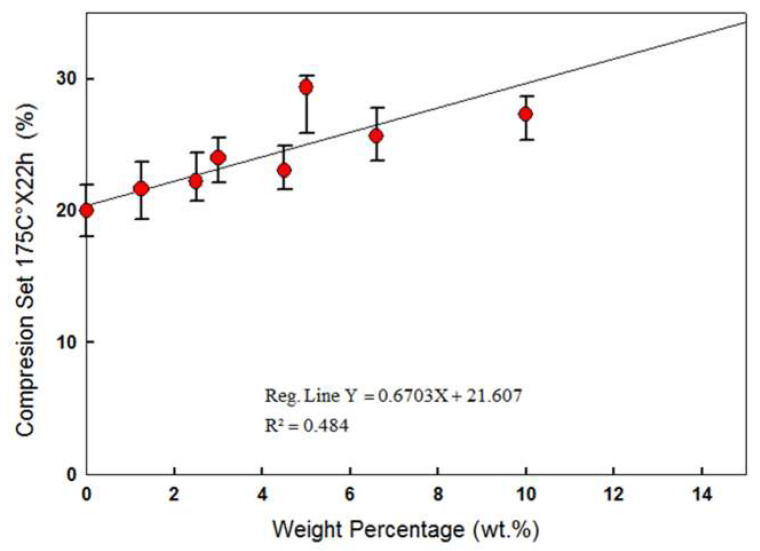
Compression Set of MWCNT/VMQ composites as a function of CNT concentration. The effect of higher than 3 wt.% CNT on Compression Set is statistically significant, but negligible from an engineering point of view (less than 10% difference).

Under compression, CNTs exhibit a clear advantage over CB: State-of-the-art 10 wt.% CB/VMQ has a compression set—irrecoverable plastic deformation after compression according to ASTM standard D395—of 60–70%. Neat VMQ has one of the lowest compression set values over the range of all other rubbers (~20%), depending on hardness and grade. CNT/VMQ, as seen in Figure 7, shows a compression set comparable to that of a neat VMQ, if slightly elevated.

### 3.3. Self-Sensing

**Figure 8:**
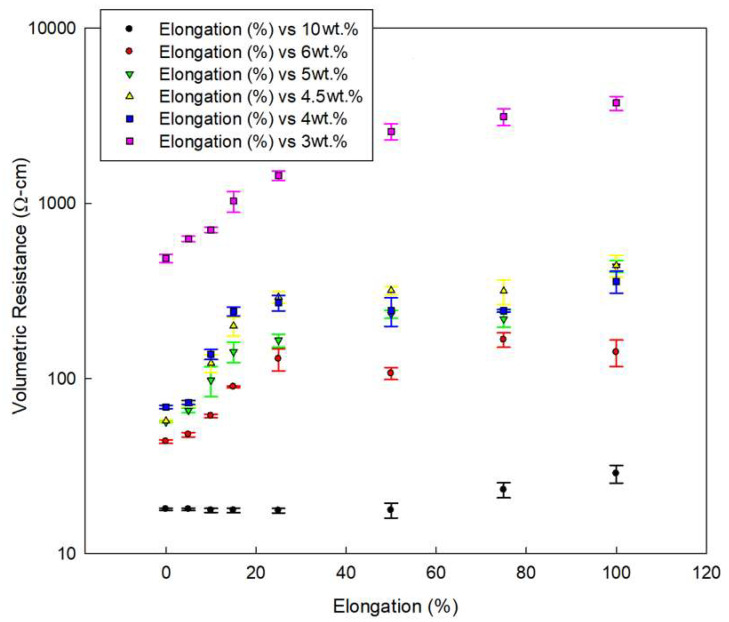
MWCNT/VMQ conductivity change with first cycle deformation, at various CNT concentrations.

Clearly seen in Figure 8 is that the resistance increases with increased extension. This observation fits previously reported work [16,17,18,30], and is usually attributed to increased MWCNT-MWCNT distance due to increased MWCNT alignment along the strain axis, alternative to the piezo-resistive nature of the MWCNT themselves [9,16,17,18,19]. Indeed, Figure 5 shows that an agglomerate aligns itself along with the strain-axis. However, Figure 5 also proposes a different mechanism to the increased resistance, i.e., discontinuity of the composite itself (and with it, obviously, the conductive network). If this is indeed the correct interpretation, then MWCNT/VMQ can not only be pressure-sensors, but also health monitoring materials.

Non-destructive Raman spectroscopy can provide information regarding the aging mechanism of the composite as a whole. MWCNTs possess strain-sensitive band shift of the 2D band. Simulating aging process can be achieved by exposure to ionizing radiation and detecting MWCNT 2D band shifts as a sensor. The 2D band position at about 2678 cm^−1^ (also known as G’), is a second-order two-phonon process, and a signature of graphitic sp2 structure. This band indicates strain conditions due to the interactions between the CNT walls [12,31]. This peak shift can indicate a good adhesion between the matrix and the CNT reinforcement, showing transfer of the radiation-induces stress from the matrix to the CNT, which causes a 2D band position shift. An upshift of the 2D band indicates that the polymer chains have applied a compressional force on the outer surface of the MWCNT, and a downshift of the 2D band indicates a tensional force. In addition, the increasing of the ratio *I*_D_/*I*_G_ (the intensities of the D band at 1345 cm^−1^ and the G band at 1580 cm^−1^, respectively) is attributed to the increasing presence of sp3 hybridization of the carbon. Such an increase therefore indicates the disruption of the π-bond electronic structure, which was found to grow due to the ionizing radiation, as previously reported by Guo et al. [32].

The results have indicated the benefit of using MWCNT embedded in the matrix as an in-situ fingerprint for the composite material degradation. An upshift of the 2D band was found in response to higher total ionizing radiation (TID). Thus, it indicates a good adhesion between the matrix and the reinforcing MWCNT, as well as indicating that, as the ageing process is ongoing, the MWCNTs are under compressional forces. That is, the ageing process of 10 wt.% MWCNT/VMQ leads to a lower ability of the material to further go through compression. The material is becoming “smart”, signaling its condition with time, allowing the user the right response to the material conditions, for example, replacing an O-ring ahead of time.

**Figure 9:**
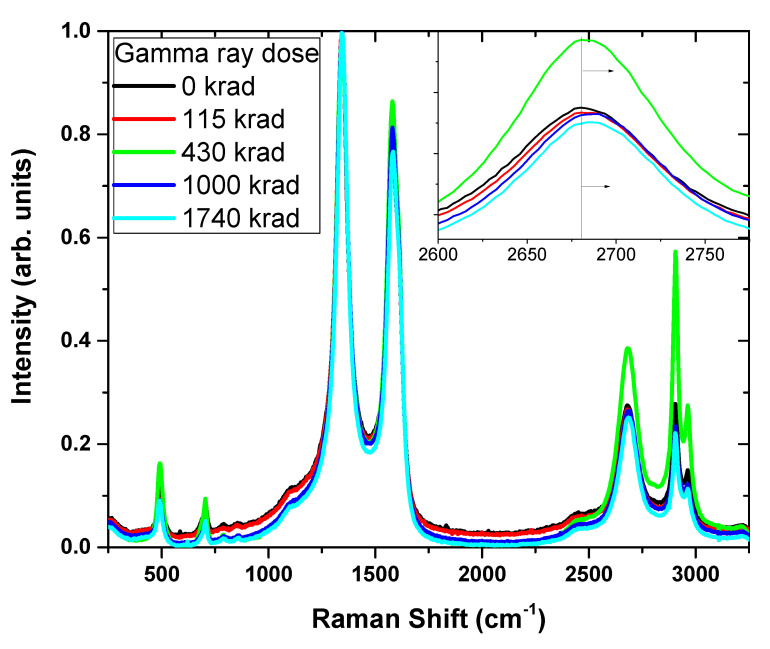
Raman spectroscopy of 10 wt.% MWCNT/Silicone rubber with gamma radiation dose. The inset is a magnification of the sensitive 2D band shift.

In order to study the ageing process of 10 wt.% MWCNT/Silicone rubber, an exposure of the samples to ionizing radiation using a ^60^Co source that produces γ-ray photons with energy in the range of 1.17–1.33 MeV was carried out with a Raman spectroscopy after each stage of radiation exposure. These Raman spectroscopy results can be further viewed in Figure 9. The maximal total absorbed dose was 1740 krad.

## 4. Conclusions

An MWCNT/VMQ composite has been successfully produced using commercial precursors and processing methods, and characterized both mechanically and electrically. The resultant composites exhibit, despite the less-than-perfect dispersion of the MWCNT agglomerates, overall lower hardness, a better compression set (less plastic deformation), and higher reproducibility than the softest CB-based conductive VMQ, with the same conductivity achieved at lower weight percentage, 4 wt.% of MWCNT, with 45 shore A vs. 10 wt.% of CB with 55 shore A.

The conductivity of MWCNT/VMQ composites was easily explained by the percolation theory adjusted to rigid rods, indicating that MWCNT–MWCNT contact is the most prominent mechanism for the transfer of electrons. Moreover, changes in conductivity due to strains can be explained mostly by changes in the overall network along the composite, more than by the piezoresistivity of the MWCNTs themselves.

The aging process of 10 wt.% MWCNT/VMQ was investigated using Raman spectroscopy. It was found that, upon exposure to TID, the samples posesses an upshift in the 2D band position, indicating a good adhesion between the matrix and the reinforcing MWCNT, as well as an increase in the compressional forces of the MWCNT. Hence, the reinforcing MWCNTs can serve as a bulk sensor regarding the internal strain conditions of the material. Both the electrical resistivity and the Raman measurements allow the soft, conductive rubber to function also as a “smart material”, self-monitoring its macro and micro stresses.

A good design of a sensor should account all of these properties, as strain-range and mechanical flexibility will also determine the sensor working range with the well-studied strain-resistivity.
